# Supplementary material for: Resident Odor Reports and Differing Health Outcomes in Areas of Industrial Emission Odor, Louisville, Kentucky
Source: medRxiv. 2025 May 15:2025.05.13.25327517. Preprint. [Version 2] doi: 10.1101/2025.05.13.25327517 (PMC12132139; doi:10.1101/2025.05.13.25327517)
Supplement: 1 [file NIHPP2025.05.13.25327517V2-supplement-1.pdf]

medRxiv preprint doi: https://doi.org/10.1101/2025.05.13.25327517; this version posted May 15, 2025. The copyright holder for this preprint (which was not certified by peer review) is the author/funder, who has granted medRxiv a license to display the preprint in perpetuity.
It is made available under a CC-BY 4.0 International license .

Supplementary Material

Resident Odor Reports and Differing Health Outcomes in Areas of Industrial Emission Odor, Louisville, Kentucky

Angelina Rangel[1], Lauren B. Anderson[1], Rochelle H. Holm[1*], and Ted Smith[1]

[1]Center for Healthy Air, Water and Soil, Christina Lee Brown Envirome Institute, School of Medicine, University of Louisville, Louisville, KY, USA

*Address correspondence to Rochelle H. Holm, Center for Healthy Air, Water and Soil, Christina Lee Brown Envirome Institute, School of Medicine, University of Louisville, 302 E. Muhammad Ali Blvd., Louisville, KY 40202 (e-mail: rochelle.holm@louisville.edu)

medRxiv preprint doi: https://doi.org/10.1101/2025.05.13.25327517; this version posted May 15, 2025. The copyright holder for this preprint (which was not certified by peer review) is the author/funder, who has granted medRxiv a license to display the preprint in perpetuity.
It is made available under a CC-BY 4.0 International license .

Table of Contents

Supplementary Table S1. Code analysis for reported odors from the Smell MyCity app. This study was limited to odor descriptions related to industrial and chemical odors (ID 2)............... 15

medRxiv preprint doi: https://doi.org/10.1101/2025.05.13.25327517; this version posted May 15, 2025. The copyright holder for this preprint (which was not certified by peer review) is the author/funder, who has granted medRxiv a license to display the preprint in perpetuity.
It is made available under a CC-BY 4.0 International license .

Supplementary Table S1. Code analysis for reported odors from the Smell MyCity app. This study was limited to odor descriptions related to industrial and chemical odors (ID 2).

ID	Resident-reported odor detail
1	Biological Mixture (fecal, rancid, decay/death, JBS)
2	Chemical Mixture (plastic, glue, rubber, sweet, polish remover, burnt/burning, garlic, cat pee/ammonia/urine, acid, industrial, chemical, alcohol)
3	Natural Gas/Fuel (gas, oil, fuel, cabbage, rotten eggs, sulfur)
4	Sewage (MSD, sewage, feces)
5	Misc (garbage, trash, open burns, drugs, etc.)
